# Long-term functional kleptoplasty in benthic foraminifera

**DOI:** 10.1016/j.isci.2025.112028

**Published:** 2025-02-14

**Authors:** Doron Pinko, Dewi Langlet, Olha Sur, Filip Husnik, Maria Holzmann, Maxim Rubin-Blum, Eyal Rahav, Natalia Belkin, Michal Kucera, Raphaël Morard, Uri Abdu, Alexander Upcher, Sigal Abramovich

**Affiliations:** 1 Department of Earth and Environmental Science, Ben-Gurion University of the Negev, Beer Sheva 8410501, Israel; 2 Okinawa Institute of Science and Technology, Evolution, Cell Biology, and Symbiosis Unit, 1919-1 Tancha, Onna-son, Okinawa 904-0495, Japan; 3 University Brest, Ifremer, BEEP, F-29280 Plouzané, France; 4 Department of Genetics and Evolution, University of Geneva, Quai Ernest Ansermet 30, 1211 Geneva, Switzerland; 5 National Institute of Oceanography, Israel Oceanographic and Limnological Research, Haifa 3102201, Israel; 6 MARUM-Center for Marine Environmental Sciences, University of Bremen, 28359 Bremen, Germany; 7 Department of Life Science, Ben-Gurion University of the Negev, Beer Sheva 8410501, Israel; 8 Ilse Katz Institute for Nanoscale Science & Technology, Ben-Gurion University of the Negev, Beer Sheva 8410501, Israel

**Keywords:** Aquatic biology, Earth sciences, Marine organism, Microorganism

## Abstract

Foraminifera are highly diverse rhizarian protists, with some lineages having developed the ability to retain chloroplasts from algal prey (kleptoplasty). Recently, we revealed the evolutionary relationship between kleptoplasty and algal symbiosis in the benthic foraminifera *Hauerina diversa*. In this study, we explored fundamental aspects of host-kleptoplast interactions. The photosynthetic rates of *H. diversa* show the sequestered kleptoplast activity under a wide range of light intensities with no signs of photoinhibition. This lack of photoinhibition response may be attributed to the loss of key elements responsible for this process during the acquisition of kleptoplasts. Our study demonstrates the stability and notably extended retention of kleptoplasty in *H. diversa*, evidenced by its plastid retention under conditions of heterotrophic feeding deprivation for 50 days. The host-kleptoplast interactions suggest that *H. diversa* is highly committed to this partnership and that this kleptoplasty species likely relies on similar kleptoplast/alga maintenance mechanisms as symbiont-bearing foraminifera.

## Highlights

• The kleptoplasts were retained in *H. diversa* for more than 50 days
• The kleptoplasts are efficient under high light with no signs of photoinhibition
• Starvation results in growth inhibition, similar to algal-bearing foraminifera
• The host is committed to maintaining the kleptoplasts under starvation stress

## Graphical abstract

**Figure:**
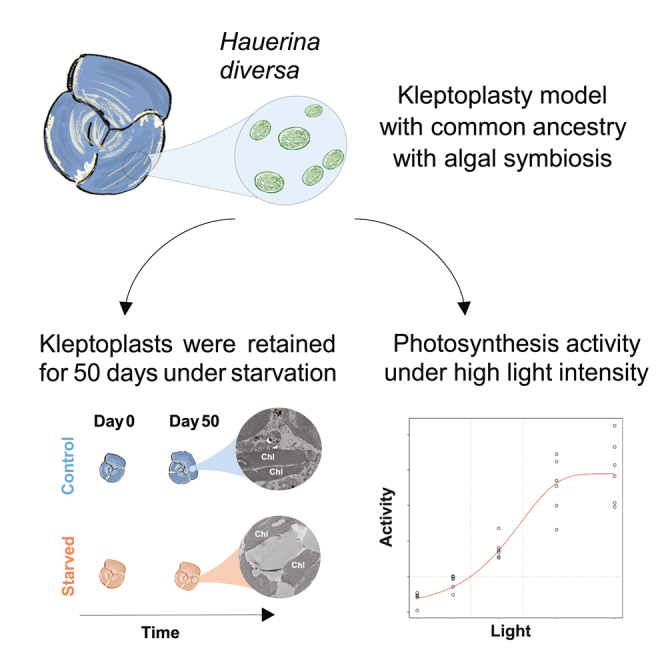

## Introduction

Endosymbiosis is a major driving force of eukaryotic ecology and evolution.^1^^,^^2^^,^^3^ An important type of endosymbiosis is “acquired phototrophy,” in which heterotrophs host algal endosymbiont or their plastids, enabling them to form mixotrophic holobionts.^4^^,^^5^ Acquired phototrophy models can differ in their stability, including a range of interactions from facultative to obligate.^4^ In the case of plastid retention, also known as “kleptoplasty,” the persistence of the interactions is puzzling since the acquired plastids have reduced genomes and are originally supported by hundreds to thousands of algal nuclear-encoded genes that are missing in heterotrophs.^6^

Kleptoplasty occurs in diverse eukaryotic lineages, including foraminifera,^7^ ciliates,^8^ dinoflagellates,^9^ centrohelids,^10^ euglenozoans,^11^ sacoglossan sea slugs,^12^ and marine flatworms.^13^ It has evolved independently several times within some lineages such as dinoflagellates,^14^ Sacoglossa,^15^ and foraminifera.^16^ The iterative evolutionary process of kleptoplasty emphasizes the ecological advantage of this life strategy. The outcome of the acquisition of chloroplasts by a predator has several main hypothesized rationales. Johnson described kleptoplasty as a parallel to stable long-term algal endosymbiosis.^4^ Alternatively, kleptoplasty has been described as an evolutionary tipping point toward an endosymbiotic transformation of a plastid to an organelle. In this case, kleptoplasty is characterized as a trial-and-error process that facilitates organelle evolution models such as the “shopping bag hypothesis” or, more broadly, the “targeting early” models.^17^^,^^18^ Nevertheless, the various kleptoplasty models that might be seen as transitional stages should not be simply viewed as leading to one endpoint of full plastid integration, but they can be considered parallel scenarios.^17^ In light of this perspective, different authors describe the study of kleptoplasty as a key to a better understanding of processes involving the acquisition and evolution of plastids.^10^^,^^19^^,^^20^

After the initial ingestion, the kleptoplasts (“stolen” plastids) are maintained under irreversible photodamage, changes in somatic pressure, and the lack of nuclear-encoded genes,^21^ ultimately determining their retention time. In Sacoglossa, it has been shown that kleptoplasts can have short retention time (RT) of hours to several days or long RT of weeks to a few months, as exhibited by different taxa.^15^ Similarly, kleptoplastidic foraminifera can exhibit short RT from only a few hours to days, as in *Ammonia tepida*,^22^ or long RT from weeks to several months, as in the case of *Elphidium williamsoni*.^23^^,^^24^ Exceptionally long RT can be found in the dinoflagellate isolated from the Ross Sea, with kleptoplast retention of 29.5 months.^25^ The maintenance of the long RT (11 weeks^26^) kleptoplasty in the ciliate *Mesodinium rubrum* is facilitated by the sequestration of the algal prey nucleus and transcriptional control.^19^^,^^27^^,^^28^

In the case of sequestration of only isolated plastids, there is a controversial hypothesis that endosymbiotic gene transfer (EGT) from the phototroph nucleus may occur to maintain the kleptoplasts.^21^ However, a growing body of evidence supports that horizontal gene transfer (HGT) from diverse donors plays a more dominant role.^29^ The host genes of foreign origin (in addition to native host genes) can then support the kleptoplast functioning via protein targeting. In Sacoglossa, the idea of EGT/HGT was rejected since, in multicellular animals, such gene transfers are largely limited by the germ line.^12^^,^^30^ Evidence of gene transfer and protein targeting was found in some protist hosts such as the centrohelid *Meringosphaera* and the Antarctic Ross Sea dinoflagellate.^10^^,^^17^ In foraminifera, data on EGT/HGT and protein targeting remain too scarce for drawing strong conclusions.^31^

Another important component in plastid maintenance is photoprotection mechanisms. These mechanisms can act at the host-behavioral level by retracting plastids away from extreme light exposure.^15^^,^^23^ It can also act at the molecular level by the maintenance of an active xanthophyll cycle, a short-term photoprotective mechanism,^32^ or a more oxidized plastoquinone pool that can suppress the formation of reactive oxygen species that would harm the plastids.^33^

Kleptoplasty in foraminifera is unequivocally diverse. It is observed from extremely deep, aphotic, and anoxic to shallow, photic, fully oxygenated environments.^7^^,^^34^^,^^35^^,^^36^^,^^37^^,^^38^^,^^39^ Kleptoplastidic foraminifera were first exclusively described in the order Rotaliida.^7^ This was later expanded by the discovery of kleptoplasty in *Hauerina diversa*, which belongs to another main foraminifera order, Miliolida.^16^ A recent study also documented kleptoplasty in the planktonic foraminifera species *Neogloboquadrina pachyderma*.^40^ The photosynthetic activity of the kleptoplasts ranges depending on the species from stable phototrophic activity for a few days to no activity.^22^ In the case of kleptoplasty in aphotic habitats, other functions were suggested, such as the uptake of nitrogen and sulfur.^41^^,^^42^ However, these findings are based mostly on the more well-known kleptoplastidic rotaliid species and emphasize the need to explore the newly identified kleptoplasty species further.

In a previous study, we discovered kleptoplasty in the foraminifera *H. diversa*, which involves a unique evolutionary linkage to algal symbiosis. This kleptoplastidic foraminifera is a sister clade to the diatom-bearing Alveolinidae. These two lineages share a closely related diatom source for both symbiosis and kleptoplasty, from a clade within Coscinodiscophycea, a group of centric diatoms.^16^ *Hauerina diversa* kleptoplasty might be seen as a transition step toward the integration of algal symbionts. However, their fossil record indicates that the kleptoplasty lineage (Hauerinidea) evolved in the Eocene ∼50 My later than the symbiont-bearing lineage (Alveolinidae).^43^ This suggests that the selection to interact with a specific diatom endosymbiont might be a synapomorphic feature of their shared ancestor.^16^

In this study, we investigate the longevity and photosynthetic functionality of *H. diversa*’s kleptoplasts following their ingestion. To assess kleptoplast longevity, *H. diversa* were cultured under food-deprived conditions to prevent the replenishment of stored kleptoplasts. The phototrophic functionality of *H. diversa*’s kleptoplasts was evaluated in a separate experiment through oxygen measurements across a range of light intensities. We reveal that this kleptoplasty model, with kleptoplasts related to algal endosymbionts, exhibits both high kleptoplast functionality and a strong commitment to kleptoplast maintenance. This suggests highly valuable photobiological advantages associated with the selected plastids.

## Results

### The kleptoplast photosynthetic activity under light gradient

**Figure 1:**
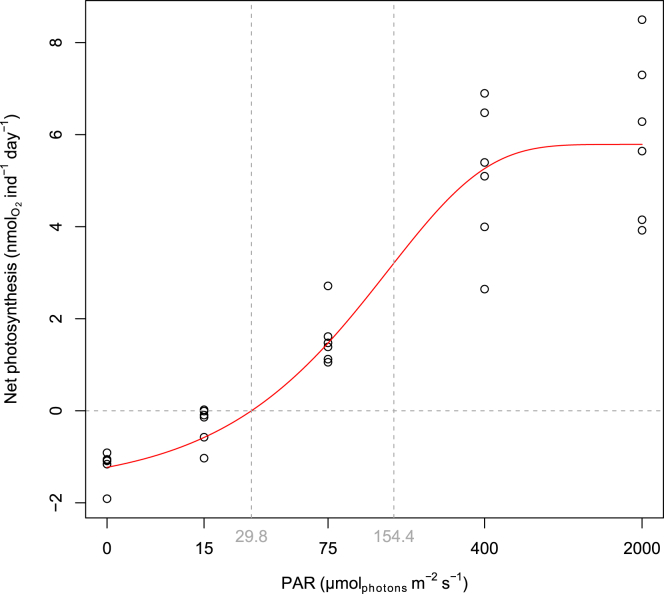
*Hauerina diversa* net photosynthesis, measured as net photosynthesis under increasing light intensities: 0, 15, 75, 400, and 2,000 PAR (μmol photons m^−2^s^−1^) Vertical dashed lines represent the compensation point (29.8 μmol photons m^−2^s^−1^) and the light saturation parameter Ek (154.4 μmol photons m^−2^s^−1^). Note the lack of photoinhibition response during high light levels.

Under increasing light levels, from darkness to high intensity of photosynthetically active radiation (PAR) 2,000 μmol photons m^−^^2^s^−1^, the net photosynthesis of the kleptoplasts revealed a steady logarithmic increase in activity, with light intensity reaching up to a maximum of 8.4 nmol/ind/day (Figure 1). The average net photosynthesis in the dark was −1.20 ± 0.36 nmol/ind/day, while it reached 5.97 ± 1.78 nmol/ind/day at 2,000 μmol photons m^−2^s^−1^. The modeled light response curve followed the equation as shown:

$${PS}_{net}=0.04*154.40*\left(1-e^{\frac{-PAR}{154.40}}\right)-1.20$$

The model predicted that the light compensation point (light intensity at which dark respiration compensates the raw photosynthesis) was reached at 29.8 μmol photons m^−2^s^−1^. Unexpectedly, the net photosynthesis to light intensity curve remains high also under high light (2,000 μmol photons m^−2^s^−1^).

### Kleptoplast retention experiment

#### Heterotrophic starvation negatively affects foraminiferal growth

**Figure 2:**
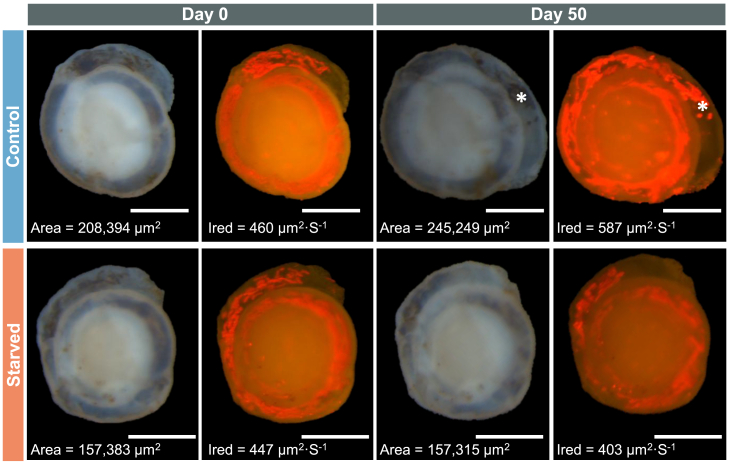
Specimens of *H. diversa* were documented during the retention experiment under a bright field and under excitation at 480 nm, triggering chlorophyll *a* autofluorescence Ired levels and foraminiferal shell area are indicated for fluorescence and bright-field images, respectively. The scale bar is 200 μm.

**Figure 3:**
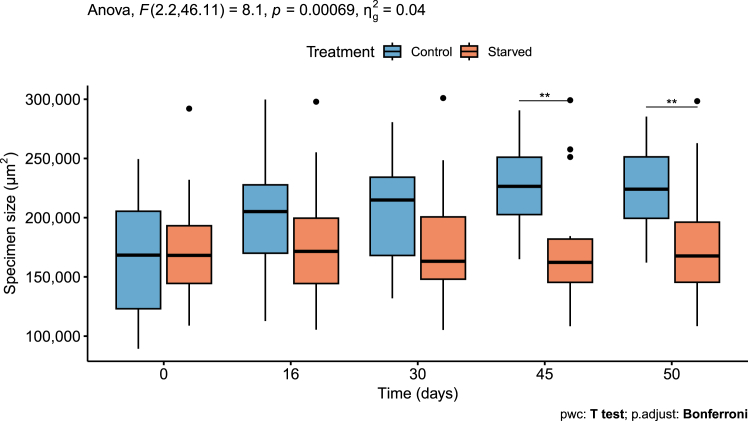
Foraminiferal size (μm^2^) is measured as specimen surface area at each time point (0, 16, 30, 45 and 50 days) Specimens cultured in natural seawater (control) showed an increase in size, indicating growth, while specimens in filtered seawater (starved) showed no growth. Data are presented in boxplot: thick horizontal lines display median values, whiskers represent maximum and minimum values, and black dots are outliers. The number of specimens in each treatment is 11 for control and 14 for starved; two extreme values are shown in the graph and were excluded from the statistical analysis (see Document S1). The mixed-design ANOVA results (top left) show a significant interaction between the treatment and time factors. A pairwise comparison between treatment levels shows a significant change in specimen size from day 45 (*p* < 0.01), as indicated by two asterisks.

At the beginning of the experiment, both treatments showed similar specimen sizes (Figures 2 and 3). Mixed-design ANOVA indicated a significant interaction between treatment and time (*p <* 0.001, Table S6; Figure 3). A pairwise comparison between treatments at different time points indicated a significant difference between control and starved specimens at >45 days (*p <* 0.01, Table S8; Figure 3), with bigger specimen sizes for the control treatment, showing a negative effect of starvation on *H. diversa* growth.

#### Long and stable kleptoplast presence in *H. diversa*

**Figure 4:**
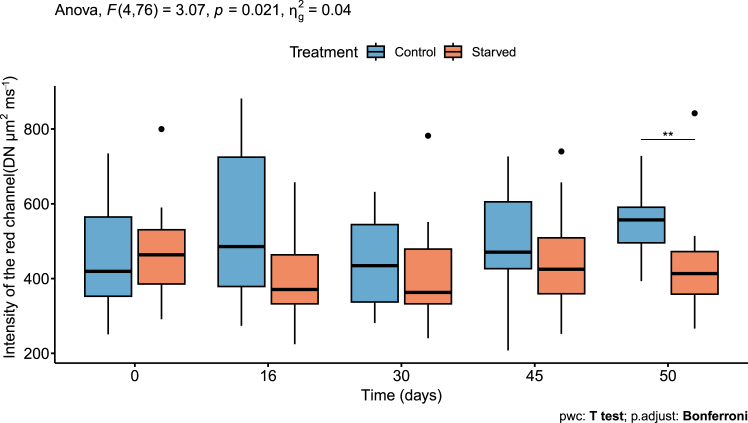
Intensity of the red channel is used as a proxy for kleptoplast presence The red fluorescence levels of specimens cultured in both treatments, natural seawater (control) and filtered seawater (starved), show similar levels up to day 45. Data are presented in boxplot: thick horizontal lines display median values, whiskers represent maximum and minimum values, and black dots are outliers. The number of specimens in each treatment is 10 for control and 12 for starved, one extreme value is shown in the graph and was excluded from the statistical analysis (see Document S1). The mixed-design ANOVA results (top left) show a significant interaction between the treatment and time factors. A pairwise comparison between treatment levels shows a significant change in red fluorescence on day 50 (*p* < 0.01), as indicated by two asterisks.

The presence of kleptoplasts within *H. diversa* during the experiment was determined by their fluorescence and is reported as intensity of the red channel (Ired) levels at each time point (0, 16, 30, 45, and 50 days, Figure 4). Generally, since Ired values are measured only for the cell surface and due to the dynamic position of the kleptoplasts in respect to the cell surface, the values of these Ired measurements vary considerably. Mixed-design ANOVA showed a significant interaction between treatment and time (*p* < 0.05, Table S18; Figure 4). A pairwise compfarison between treatments at different times indicated significantly lower Ired levels of starved compared to control specimens at day 50 (*p* < 0.01, Table S20; Figure 4), but not before. Moreover, the post hoc test shows that in both control and starved treatments, the fluorescence levels remained stable and did not decline over time when testing differences between time points (Table S22).

#### Ultrastructure of *H. diversa* specimens under starvation

**Figure 5:**
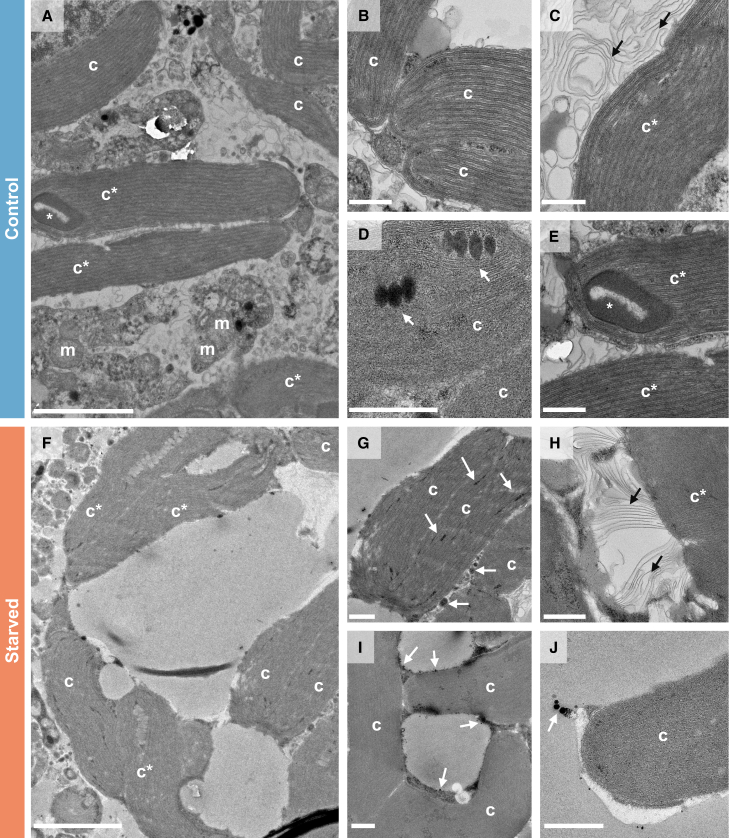
Diatom chloroplasts found in *H. diversa* cytoplasm as shown in TEM micrograph of specimens cultured for 50 days (A–E) were cultured in natural seawater (control) and (F)–(J) in filtered seawater (starved). (A and F) Chloroplast clusters, including intact chloroplasts (c) and partially degraded chloroplasts (c∗), note the presence of mitochondria (m) confirming the well-being of the foraminiferal specimen. (B and G) Intact chloroplasts reveal internal structures composed of thylakoid membranes. (C and H) Degraded chloroplasts with loose thylakoid membranes. (D and I) Lipid droplets within a cluster of kleptoplasts. (E) Pyrenoid partially degraded (white asterisk). (J) Lipid droplets are released from the kleptoplasts to the host cytoplasm. White arrows point to lipid droplets. Black arrows point at the degraded thylakoid membranes. The scale bar is 2 μm for (A) and (F) and 500 nm for (B)–(E) and (G)–(J).

The transmission electron microscopy (TEM) micrographs of specimens from the retention experiment revealed abundant kleptoplasts after 50 days in both starved and control treatments (Figures 5A and 5F). In both treatments, some intact kleptoplasts were observed (Figures 5B and 5G), while others showed signs of degradation, evident by loose thylakoid membranes and damaged pyrenoid (Figures 5C, 5E, and 5H). Another common feature of the kleptoplasts was the presence of black bodies recognized as lipid droplets, which are known features of diatom chloroplasts.^44^ These droplets were observed within the kleptoplasts (Figures 5D and 5I) and were also documented released from the kleptoplasts to the host cytoplasm (Figure 5J).

## Discussion

Despite extensive research on a few kleptoplasty systems, much remains unexplored in many recently discovered kleptoplastidic species, such as the unique case of *H. diversa*. Establishing new kleptoplasty models is thus essential to expand our understanding of the evolution of acquired autotrophy.^9^^,^^10^ In this study, we have explored the recently discovered kleptoplasty in *H. diversa*, targeting fundamental elements of the interaction, such as the RT and the functionality of the kleptoplasts.

### Kleptoplasts in *H. diversa* are photosynthetically active

The net photosynthesis measurements reveal that the kleptoplasts in *H. diversa* are photosynthetically active under a wide range of light intensities (25–2,000 μmol photons m^−2^s^−1^). This functionality was previously also identified in other kleptoplastidic foraminifera species. In comparison to the two most studied kleptoplasty foraminifera, the net photosynthesis of *H. diversa* at 400 μmol photons m^−2^s^−1^ (Figure 1) is about 5 times larger than those reported at similar light intensities in *Haynesina germanica*,^22^^,^^45^^,^^46^ but similar to *E. williamsoni*.^23^ The modeled light response curve shows that the light compensation point for *H. diversa* is at 29.8 μmol photons m^−2^s^−1^. This value is similar to that reported in *H. germanica* (24 μmol photons m^−2^s^−1^).^22^ Both *E. williamsoni* and *H. germanica* inhabit intertidal mudflat environments with wide-ranging light intensities. However, *E. williamsoni* has a lower compensation point (5.6 μmol photons m^−2^s^−1^),^23^ which might be an adaptation to lower light conditions compared to *H. germanica* and *H. diversa*.^23^

The photosynthetic saturation constant (E_k_) for *H. diversa* was 154.4 μmol photons m^−2^s^−1^, which is higher than the values reported for both *H. germanica* and *E. williamsoni* (between 17 and 90 μmol photons m^−2^s^−1^).^22^^,^^23^^,^^38^ Generally, a higher saturation constant (E_k_) can be attributed to an adaptation to higher light intensities.^47^ This can be explained by the habitat differences between *H. diversa*, which lives on macroalgae as an epiphytic species, compared to *H. germanica* and *E. williamsoni*, which inhabit the top millimeters of sediments. Another factor that might contribute to these differences is that *H. diversa* has a porcelaneous, opaque shell as a miliolid, while *H. germanica* and *E. williamsoni* are rotaliids with hyaline, transparent shells. These shell types might influence the amount of light reaching the kleptoplasts, allowing *H. diversa* to tolerate higher light intensities.

The summer light intensity in *H. diversa*’s habitat is reaching >2,000 μmol photons m^−2^s^−1^, which can explain its high performance under these high light intensities (Figure 1). The logarithmic increase in activity with a high light intensity of up to 2,000 μmol photons m^−2^s^−1^ might also indicate that this system lacks some photoinhibition mechanisms, which would shield the light-harvesting system from excess and harmful light levels. The lack of photoinhibition response was also documented in the kleptoplastidic foraminifera *E. williamsoni*^23^ and *H. germanica*.^22^ A possible explanation for the lack of photoinhibition response might be the partial loss of key elements involved in this process during the kleptoplast acquisition.

### *H. diversa* retains kleptoplasts for over 50 days

Our goal was to establish whether *H. diversa* is a short- or long-term kleptoplastidic host. This is noteworthy since the duration of the RT has major implications for host-kleptoplast interactions. Indeed, short-term kleptoplasty was hypothesized to be used for more immediate functions such as food storage and crypsis.^48^^,^^49^ Longer RT is attributed to a higher phototrophic function^38^ and requires some maintenance to ensure the kleptoplasts’ survival.^21^

This study reveals that the RT in *H. diversa* was notably long and exceeded the time frame of the experiment. Therefore, we can only determine that the RT of the kleptoplasts is longer than 50 days. This was verified by the kleptoplasts’ autofluorescence (Figure 4) and TEM imaging (Figure 5) of specimens cultured under starvation to eliminate the possibility of kleptoplast replenishment. Since the survival of kleptoplasts sequestered by *H. diversa* exceeds 1 month, it can be described as long-term kleptoplasty following the definition of Händeler et al.^15^ Long RT was also described in the kleptoplastidic foraminifera *E. williamsoni*, which showed under starvation a major decrease in chlorophyll within 20–30 days and a complete reduction at ∼60 days.^24^ In contrast, the rate of kleptoplasts’ degradation in *H. diversa* is extremely low, with only partial degradation after 50 days of starvation, indicating that the potential RT can be much longer. This observation indicates active kleptoplast protection by the host or selective acquisition of a diatom species with an exceptionally robust plastid.^21^

### Why and how does *H. diversa* maintain the kleptoplasts long term?

The stability of *H. diversa* kleptoplasty was implied by its existence in all specimens collected from the field and by its long-term prevalence within the host during the starvation experiment. Surprisingly, no significant change in kleptoplast presence was observed between the starved and the control treatments until the last measurement at 50 days (Figure 4). This similarity may stem from the low abundance of benthic diatoms in the control (natural seawater) treatment, as *H. diversa* diatom prey might reside mostly in the substrate rather than in the seawater.

The kleptoplasts’s presence in the starved (filtered seawater) treatment, lacking any food sources, indicates that the host is committed to maintaining the kleptoplasts even under the stress of ongoing starvation. This is further supported by the growth inhibition shown in the starved treatment. It demonstrates that the foraminiferal host did not digest its abundant kleptoplasts to compensate for the lack of nutrients. Instead, the host response observed was the inhibition of high-energy functions of calcification and growth. This observation implies that *H. diversa* relies on heterotrophic feeding to support activities with high-energy requirements, as also documented in algal symbiont-bearing foraminifera.^50^ Although *H. diversa* acquires autotrophy by kleptoplasty, it cannot fully function as an autotroph and must rely on heterotrophy for growth, similar to algal symbiont-bearing foraminifera.

The presence of released lipid droplets from the kleptoplasts observed by TEM (Figure 5J) suggests an active supply, possibly stress-induced, of nutrients from the kleptoplasts to the starved host. It also provides additional evidence that kleptoplasts are likely active even after >50 days from their initial acquisition. This feature was also previously observed in two other kleptoplastidic foraminifera species: *E. williamsoni* and *H. germanica*.^23^^,^^51^ It was suggested that the lipids produced by the kleptoplasts are used as an additional carbon source for the host and that their production is enhanced under stress conditions such as starvation.^23^ The contribution of the kleptoplasts to survival during times of starvation was also extensively studied in sea slugs.^21^ Yet, in a study that tested both the effect of feeding and light, both had a positive effect on survival, demonstrating that the kleptoplasty function during food scarcity cannot completely compensate for starvation.^52^

Our results show that kleptoplasty in *H. diversa* is stable and notably long. Long RT has major implications for the interactions of the host with the kleptoplasts, implying that kleptoplasty is not a transitional or temporal phase but a long-lasting interaction with some similarities to long-term algal symbiosis. We hypothesize that the shared ancestry of *H. diversa* to an algal symbiont-bearing foraminiferal host might be the key to revealing the mechanisms that allow this stable kleptoplasty.

### Limitations of the study

In this study, we provided evidence of photosynthetic activity in *H. diversa*, which was achieved by the oxygen experiment. Our results do not rule out the presence of photoinhibition mechanisms, but we also cannot confirm their efficiency, as no evidence of photoinhibition was observed. Moreover, the long retention time was documented using autofluorescence to provide evidence of the presence and abundance of chlorophyll *a*, and hence kleptoplasts. However, autofluorescence does not indicate the functionality of the retained kleptoplasts, and this functionality should be further tested in the future.

## Resource availability

### Lead contact

Requests for further information and resources should be directed to and will be fulfilled by the lead contact, Sigal Abramovich (sigalabr@bgu.ac.il).

### Materials availability

This study did not generate new unique reagents.

### Data and code availability

• All the raw data of the retention experiment is provided in the Document S1.
• Raw data and calculation pipeline for the photosynthesis experiment are publicly accessible at Zenodo: https://doi.org/10.5281/zenodo.14768417.
• Any additional information will be provided upon request.

## Acknowledgments

We are grateful to Gosia Broszkiewicz from Okinawa Institute of Science and Technology (OIST) for her technical support of the TEM sample preparation. We would also like to thank Nir Ben-Eliahu for his advice and support in the autofluorescence analysis. Funding was provided to D.P. by the Cushman Foundation for Foraminiferal Research, the Mediterranean Sea Research Center of Israel, and the Adams Fellowships Program of the Israel Academy of Sciences and Humanities. F.H. was supported by 10.13039/501100001691JSPS KAKENHI grant 23K14256 and the HFSP Early Career Grant (RGEC29/2024). D.L. was supported by 10.13039/501100001691JSPS KAKENHI grant 23K05942. S.A. was supported by the 10.13039/501100003977Israel Science Foundation grant no. 941/17.

## Author contributions

D.P. performed the experimental studies and carried out the analysis, conceptualization, and writing. D.L. performed experimental studies, carried out the net photosynthesis analysis, and writing. O.S. contributed to the net photosynthesis experiment. F.H.: supervision, writing, and editing. M.H., M.R.B., E.R., N.B., M.K., and R.M.: writing and editing. A.U. contributed to the electron microscopy work. U.A. and S.A.: supervision, conceptualization, writing, and editing.

## Declaration of interests

The authors declare no competing interests.

## STAR★Methods

### Key resources table

**Table:** 

REAGENT or RESOURCE	SOURCE	IDENTIFIER
**Chemicals, peptides, and recombinant proteins**		

Uranyl acetate	SPI	CAS#6159-44-0

**Deposited data**		

Retention experiment- raw data	This paper	Document S1
Photosynthesis experiment- raw data and calculation pipeline	This paper	Zenodo: https://doi.org/10.5281/zenodo.14768417

**Experimental models: Organisms/strains**		

*Hauerina diversa* specimens	Two sites, Akhziv (33°03'55.5″*N* 35°06'16.8″E) and Shikmona (32°49'33.6″*N* 34°57'20.8″E)	N/A

**Software and algorithms**		

ImageJ	NIH	https://imagej.net/ij/
R v 4.0	R Core Team	https://www.r-project.org/

**Other**		

Transmission Electron Microscope	ThermoFisher Scientific	Talos F200C
Stereomicroscope	Leica	M165 FC
Quantum meter	Apogee instruments	MQ-200
Oxygen microelectrode	Unisense	N/A
Laboratory microprofiler	Unisense	N/A

### Experimental model and study participant details

#### Specimen collection

Specimens of *H. diversa*, a well-established Lessepsian invader introduced from the Red Sea,^53^^,^^54^ were collected from two sites, Akhziv (33°03'55.5″*N* 35°06'16.8″E) and Shikmona (32°49'33.6″*N* 34°57'20.8″E), located on the northern Mediterranean coast of Israel, where this species has a relatively high abundance, especially during the winter.^55^ Specimens were collected from the macroalgal mats covering the rocky reef environments at 20–40 cm water depth. These two sites are natural reserves, and the collection of samples was done under a special permit (No. 2024/43688) to collect wildlife given by the Israel Nature and National Parks Protection Authority.

#### Specimen care and maintenance

Living and healthy adult specimens were picked under a stereomicroscope (Leica M165 FC) and distinguished from dead specimens based on their dark cytoplasmic color and reticulopodial activity observed after several hours. Additionally, kleptoplasts presence was indicated by the detection of choloropyll *a* using a stereomicroscope with Fluorescence Illumination LED Light Source (Leica SFL100). After the initial collection process, specimens were kept in natural seawater under 27°C and 12-h light:12-h dark cycle (10–15 μmol photons m^−2^s^−1^) until they were transferred for further analysis.

### Method details

#### Photosynthetic response to light of *H. diversa*

The photosynthesis experiment was performed to examine the functionality of the kleptoplasts by measuring their oxygen production at different light intensities. Three *H. diversa* specimens were placed at the bottom of a glass micro-tube (10 mm in height, 1 mm inner diameter) under increasing light levels (0, 15, 75, 400, 2000 μmol photons m^−2^s^−1^ provided by a Nikon C-FLED2 light source and controlled with an MQ-200 quantum meter, Apogee instruments). These high light levels can be found in the sampling site, with summer light intensity reaching >3000 μmol photons m^−2^s^−1^ on the surface and ∼1500 μmol photons m^−2^s^−1^ at 40 cm depth as indicated by field measurements from Shikmona, June 2024. The oxygen gradients in the millimeter above the foraminifera were recorded using a 50 μm tip diameter microelectrode (Unisense, sensor 2-point calibrated following the manufacturer’s protocol). Under each light condition, 5 to 15 vertical microprofiles were realized at a 100 μm resolution using a laboratory microprofiler (Unisense) to ensure that oxygen consumption/production reached a steady state (as indicated by the parallelism of oxygen profiles). The examined specimens were exposed to each tested light level for a short time of a few minutes until reaching a steady state.

A total of 6 replicates were measured, each consisting of sets of 3 specimens exposed to 5 light intensities. All measurements were performed in micro-filtered (<0.22μm) natural Mediterranean seawater (salinity = 38psμ) in a temperature-controlled room. To ensure temperature stability throughout the experiment, the measurement microtubes were placed in a 6L aquarium (leading to water temperatures ranging from 22.22°C to 22.36°C). Individual oxygen photosynthesis rates were calculated from each oxygen profile following Deldicq et al.^45^ The light response curve was fitted following the Webb model^56^ bellow:

$${PS}_{net}=\propto *E_{k}*\left(1-e^{\frac{-PAR}{E_{k}}}\right)+{PS}_{0}$$

With *PS*_*net*_ the net photosynthesis rate, *α* the initial slope, *E*_*k*_ the light saturation parameter, *PAR* the light intensity, and *PS*_*0*_ the photosynthesis rate in the dark. The model was fitted using phytotools and FME R packages^57^^,^^58^ in R v 4.0.^59^ All the raw data and calculation pipeline are publicly accessible at https://doi.org/10.5281/zenodo.14768417.

#### Kleptoplast retention experiment

##### *Hauerina diversa* culturing under starvation

The retention experiment was performed to evaluate the longevity of *H. diversa*’s kleptoplasts using culturing under starvation compared to control conditions. Specimens collected from the field were first acclimated in an incubator for a week at a temperature of 27°C and 12-h light:12-h dark cycle with light intensity of 10–15 μmol photons m^−2^s^−1^ photosynthetically active radiation (PAR) as indicated by MQ-200 quantum meter, Apogee instruments. Forty specimens were individually placed in separate 1.5 mL centrifugation tubes; twenty with natural seawater (control) and twenty with <0.22μm filtered seawater (starved), thus devoid of any food or kleptoplast replenishment source. The natural seawater in the control treatment contains the natural population of bacteria and algae from the sampling site that can be consumed as food by the foraminifera. The specimens were cultured for 50 days under the same temperature and light conditions as during the acclimation. Every two weeks, the culture medium was replaced, and the specimens' state was documented by imaging under a bright field and fluorescent light. After 50 days, the experiment was terminated since mortality reached 37.5%, with 15 dead specimens out of 40 specimens overall, 9 in the control and 6 in the starved treatment. All specimens that died during the experiments were removed from the image analysis.

##### Image analysis

The effect of starvation on the degradation of the kleptoplasts was documented every two weeks using chlorophyll *a* autofluorescence analysis approach. The fluorescent light excitation at 480 nm was used as a proxy of chlorophyll autofluorescence. All fluorescence images were taken with fixed camera settings (exposure time of 220 ms, 0.7 gamma, and 4 gain). This method quantifies the chlorophyll *a* stimulated autofluorescence by using red channel digital numbers (DNs) in each fluorescence image. The DNs values were normalized to the exposure time and magnification. The resulting values are called ‘intensity of the red channel’ (Ired) values. Based on Friedrichs et al.^60^ Ired has a positive correlation with chlorophyll *a* concentrations and, therefore can be used for estimating the chlorophyll content in a non-invasive process.

The use of Ired as an indicator for algal symbionts' chlorophyll content in the foraminiferal host was established by Ben-Eliahu et al.,^61^ which documented a reduction in algal symbionts' chlorophyll content by both Ired analysis and a direct chlorophyll content measurement. In the case of *H. diversa*, the chlorophyll content is limited to the kleptoplasts clusters and is not consistent throughout the cell. Therefore, the Ired values can indicate the presence of chlorophyll *a*, and hence kleptoplasts abundance only at the foraminiferal surface. Following Ben-Eliahu et al., to clean unwanted DN levels, such as black background and white scale bar, extremely low (0–0.125, dark gray-black) and high (0.825–1, light gray-white) DNs were excluded from the analysis.^61^ Another level of cleaning was achieved by excluding three specimens that developed an external debris cover that might interfere with the evaluation of the internal kleptoplasts autofluorescence. These specimens were removed only from the autofluorescence analysis and not from the growth analysis.

During the retention experiment, the foraminiferal host growth was documented using size measurements of the foraminiferal specimen surface area (μm^2^) in lateral view. Changes in foraminiferal shell size indicate active growth and calcification, which require a high energy level and, therefore, can be used as a proxy of the host’s well-being. The foraminiferal size was measured using ImageJ^62^ (V 1.53t) software based on the bright field images and a digital scale bar was used for calibration.

##### Transmission Electron Microscopy (TEM)

For visual documentation of the kleptoplasts' condition after 50 days of the retention experiment, four specimens (two of each treatment) were fixed and prepared for Transmission Electron Microscopy as previously described in Pinko et al.^16^ The ultra-thin sectioning was done using Leica EM UC7 Ultramicrotome at OIST, Okinawa, Japan. Before imaging, the ultra-thin sections were stained with 2% uranyl acetate solution for 1 min. The micrographs were obtained by Talos F200C (ThermoFisher Scientific) transmission electron microscope operating at 200 kV at the Ilse Katz Institute, BGU, Israel.

### Quantification and statistical analysis

To evaluate whether the differences in specimen sizes and fluorescence levels (Ired) are significant between the factors of time and treatment, we performed statistical analysis using R v 4.0.^59^ For each dataset (specimen sizes and fluorescence levels, see Tables S1, S11, and S12), we tested the assumptions of normality of the residuals, homogeneity of variances, and homogeneity of covariances. To meet these assumptions, we first tested for extreme values and removed them from the statistical analysis (see Tables S2, S13, and S14). For both datasets, the assumptions were valid (see Tables S3–S5 and S15–S17), and a mixed-design ANOVA model was performed (see Tables S6 and S18). This ANOVA model, which includes both a between-subject factor (Treatment) and a within-subject factor (Time), was selected to consider the repeated measures of the same specimens at different time points. Since a significant interaction between the two factors was found in both datasets, the mixed-design ANOVA was followed by the proper post hoc tests to check for the effect of one factor in each level of the other factor (e.g., testing for the effect of the ‘Treatment’ factor on every level of ‘Time’ factor, see Tables S7–S10, and S19–S22). Each single specimen was evaluated as a replicate. Growth analysis had *n* = 11 for control and *n* = 14 for starved. Autofluorescence analysis had *n* = 10 for control and *n* = 12 for starved. The difference in the number of replicates is caused by three specimens that were excluded from the autofluorescence analysis (see more information in the section ‘image analysis’).

### Additional resources

All the raw data and calculation pipeline for the photosynthesis experiment are publicly accessible at https://doi.org/10.5281/zenodo.14768417.
