# Supplementary material for: Long-term functional kleptoplasty in benthic foraminifera
Source: iScience. 2025 Feb 14;28(3):112028. doi: 10.1016/j.isci.2025.112028 (PMC11926685; doi:10.1016/j.isci.2025.112028)
Supplement: Document S1. Tables S1–S22 [file mmc1.pdf]

iScience, Volume *28*

Supplemental information

Long-term functional kleptoplasty

in benthic foraminifera

Doron Pinko, Dewi Langlet, Olha Sur, Filip Husnik, Maria Holzmann, Maxim Rubin-Blum, Eyal Rahav, Natalia Belkin, Michal Kucera, Raphaël Morard, Uri Abdu, Alexander Upcher, and Sigal Abramovich

Supplementary Information- Retention Experiment statistics

Statistical analysis of specimen sizes

Table S1: raw data, values are the surface area in μm^2, related to Figure 3.

ID	Day 0	Day 16	Day 30	Day 45	Day 50
C2	249,471.20	299,732.25	280,635.80	290,596.57	285317.3
C3	247,799.98	248,441.00	256764.368	272281.394	265526.9
C5	202,406.13	203,605.47	247358.477	255079.64	257413.5
C7	89,310.62	128,512.58	132105.839	190613.559	190500.5
C12	208,393.62	205,128.23	221,044.16	247079.224	245248.9
C14	168,384.39	215,900.65	212894.98	214567.785	208444.9
C15	100,562.28	219,079.00	214926.597	216195.267	224052.8
C16	191,655.44	192,367.04	191419.752	190050.712	186725.2
C18	112,706.42	112,755.38	131915.074	226413.761	229522.7
C19	145,492.51	147,603.27	144801.22	164950.514	162055.8
C20	133,506.34	236,370.84	218256.749	228073.952	211252.2
T1	214,911.82	248,770.76	248449.436	251215.522	251019.8
T2	172,080.56	175,361.99	166484.947	174618.713	173650.2
T3	150,009.43	148,132.28	150092.882	145309.917	150168
T4	165,037.83	161,154.52	155848.905	159459.893	161760.7
T6	171,326.95	179,418.46	180143.928	173900.78	176400.1
**T7**	**292,058.63**	**297,884.98**	**300976.114**	**299175.429**	**298373.1**
T8	157,383.35	167,669.17	159859.557	165134.564	157315.2
T9	142,592.27	136,780.64	142020.226	145537.167	143935.7
T10	181,574.93	182,700.77	180791.781	184423.269	186428.9
**T11**	197,063.04	205,261.44	207229.251	**154934.49**	199430.4
T12	108,876.15	105,425.44	105222.87	108402.3	108448.7
T13	140,213.31	143,171.30	142,676.97	144784.238	141373.9
T14	133,554.58	143,098.71	147422.943	139852.955	142193.4
T17	231,928.88	255,133.19	246994.839	257652.142	262851.8

Removal of extreme and missing data points-

- Specimens that died during the 50 days of the experiment were removed.
- Values in red are extreme values.
    - **T11** day 45- is extremely low compared to all other T11 values.
    - **T7** – extremely large compared to all other specimens in all treatments and time points.

Before removing extreme values, an ANOVA test was performed on the raw data to ensure that removing these values would not change the outcome of the statistical test. Removing these extreme values was needed to fulfill the assumptions of the ANOVA test.

Table S2: Test for outliers and extreme values, related to STAR Methods.

Treatment	Time	ID	Size (µm^2)	Is.outlier	Is.extreme
T	0	T17	231929	True	False
T	16	T1	248771	True	False
T	16	T17	255133	True	False
T	30	T1	248449	True	False
T	30	T17	246995	True	False
T	45	T1	251216	True	False
T	45	T17	257652	True	False
T	50	T1	251020	True	False
T	50	T17	262852	True	False

Table S3: Normal distribution of the dependent variable tested by Shapiro-Wilk normality test, related to STAR Methods.

Treatment	Time (days)	Variable	Statistic	p
C	0	growth	0.9413931	0.53701230
C	16	growth	0.9612069	0.78665601
C	30	growth	0.9199069	0.31786538
C	45	growth	0.9822931	0.97755789
C	50	growth	0.9864253	0.99145047
T	0	growth	0.9579206	0.75378541
T	16	growth	0.8929958	0.12880594
T	30	growth	0.8665072	0.05902522
T	45	growth	0.8660394	0.05822779
T	50	growth	0.8692523	0.0639388

Table S4: Homogeneity of variance tested by Levene's test, related to STAR Methods.

df	F value	Pr (>F)
9	0. 5609393	0. 8261117
105		

Table S5: Homogeneity of covariances tested by Box's M test, related to STAR Methods.

statistic	p.value	parameter	method
2.84	0.0917	1	Box's M-test for Homogeneity of Covariance Matrices

Table S6: ANOVA mixed model, the effect of time and treatment on specimens' sizes, related to Figure 3.

Effect	DFn	DFd	F	p	P<.05	ges
Treatment	1	21.00	4.174	5.40e-02		0.148
Time	2.2	46.11	12.890	2.11e-05	*	0.070
Treatment:Time	2.2	46.11	8.095	6.94e-04	*	0.045

Post hoc tests

Effect of the treatment factor -

Table S7: Effect of the treatment factor on every level of time, related to STAR Methods.

Time	Effect	DFn	DFd	F	p	P<.05	ges	p.adj
0	treatment	1	21	0.044	0.836		0.002	1
16	treatment	1	21	2.18	0.155		0.094	0.775
30	treatment	1	21	3.49	0.076		0.143	0.38
45	treatment	1	21	10.7	0.004	*	0.338	0.02
50	treatment	1	21	9.36	0.006	*	0.308	0.03

Table S8: Pairwise comparisons between treatment levels, related to Figure 3.

Time	group1	group2	n1	n2	p	p.signif	p.adj	p.adj.signif
0	C	T	11	12	0.836	Ns	0.836	ns
16	C	T	11	12	0.155	Ns	0.155	ns
30	C	T	11	12	0.0757	Ns	0.0757	ns
45	C	T	11	12	0.00362	**	0.00362	**
50	C	T	11	12	0.00595	**	0.00595	**

Effect of the time factor-

Table S9: Effect of the time factor on every level of treatment, related to STAR Methods.

Treatment	Effect	DFn	DFd	F	P	p<.05	ges	p.adj
C	Time	2.16	21.6	10	0.000654	*	0.176	0.00131
T	Time	1.63	18	2.88	0.09		0.004	0.18

Table S10: Pairwise comparisons between time points at each treatment level, related to STAR Methods.

Treatment	group1	group2	n1	n2	p.adj	p.adj.signif
C	0	16	11	11	0.328	ns
C	0	30	11	11	0.071	ns
C	0	45	11	11	0.007	**
C	0	50	11	11	0.015	*
C	16	30	11	11	1	ns
C	16	45	11	11	0.484	ns
C	16	50	11	11	0.938	ns
C	30	45	11	11	0.306	ns
C	30	50	11	11	0.691	ns
C	45	50	11	11	1	ns
T	0	16	12	12	0.853	ns
T	0	30	12	12	1	ns
T	0	45	12	12	0.825	ns
T	0	50	12	12	0.77	ns
T	16	30	12	12	1	ns
T	16	45	12	12	1	ns
T	16	50	12	12	1	ns
T	30	45	12	12	1	ns
T	30	50	12	12	1	ns
T	45	50	12	12	1	ns

Statistical analysis of the Intensity of the red channel (Ired)

Table S11: raw data of specimens cultured in control treatment, values are DN μm^{-2} ms^{-1}, related to Figure 4.

ID	Time	Ired
C2	0	734.6429
C2	16	881.5981
C2	30	631.951
C2	45	726.5829
C2	50	727.861
C5	0	600.1549
C5	16	531.4339
C5	30	567.181
C5	45	470.4368
C5	50	608.8022
C7	0	285.3755
C7	16	287.9496
C7	30	281.0116
C7	45	463.6339
C7	50	403.7323
C12	0	459.6762
C12	16	789.2737
C12	30	414.8619
C12	45	670.0314
C12	50	587.3887
C14	0	358.817
C14	16	803.5552
C14	30	559.1656
C14	45	621.7418
C14	50	591.9623
C15	0	251.0097
C15	16	500.6163
C15	30	499.6792
C15	45	207.7081
C15	50	551.6076
C16	0	599.6612
C16	16	453.9978
C16	30	454.2409
C16	45	556.1081
C16	50	499.9675
C18	0	350.7905
C18	16	273.6354
C18	30	313.8639
C18	45	470.6091
C18	50	562.3118
C19	0	411.8154
C19	16	353.6701
C19	30	359.1652
C19	45	414.1079
C19	50	393.2234
C20	0	427.0461
C20	16	470.3283
C20	30	330.0774
C20	45	276.9891
C20	50	494.1621

Table S12: raw data of specimens cultured in starved treatment, values are DN μm^{-2} ms^{-1}, related to Figure 4.

ID	Time	Ired
T1	0	552.5911
T1	16	542.8286
T1	30	490.5493
T1	45	595.0873
T1	50	434.7255
T2	0	464.2768
T2	16	362.2453
T2	30	335.2413
T2	45	480.2825
T2	50	470.3305
T3	0	463.5791
T3	16	342.817
T3	30	332.2027
T3	45	462.4393
T3	50	361.0333
T6	0	523.1158
T6	16	432.366
T6	30	473.4868
T6	45	360.5562
T6	50	424.1373
T7	0	799.8536
T7	16	654.5343
T7	30	782.0523
T7	45	739.9886
T7	50	842.1244
T8	0	446.5858
T8	16	372.7724
T8	30	390.664
T8	45	399.968
T8	50	402.5507
T9	0	336.2229
T9	16	286.2112
T9	30	300.3946
T9	45	285.5155
T9	50	349.8169
T10	0	463.5309
T10	16	437.0989
T10	30	474.9892
T10	45	449.9993
T10	50	477.3875
T12	0	291.2624
T12	16	224.3737
T12	30	240.5301
T12	45	252.0271
T12	50	266.3856
T13	0	352.017
T13	16	301.5137
T13	30	332.5016
T13	45	368.8505
T13	50	332.2173
T14	0	396.6668
T14	16	368.7809
T14	30	334.7367
T14	45	355.8502
T14	50	361.2145
T17	0	589.9857
T17	16	657.5494
T17	30	551.1142
T17	45	657.2641
T17	50	513.9366

Table S13: test for outlier and extreme values, related to STAR Methods.

Treatment	Time	ID	Ired	is.outlier	is.extreme
T	0	T7	800	TRUE	FALSE
T	30	T7	782	TRUE	FALSE
T	45	T7	740	TRUE	FALSE
T	50	T7	842	TRUE	TRUE

Following this analysis, sample ID T7 was marked as extreme and excluded from the statistical analysis to eliminate any possible bias these values might cause. ANOVA assumptions and results were tested with and without the extreme values to ensure that removing them did not affect the statistical results.

Table S14: test for outlier and extreme values, after removal of sample T7, related to STAR Methods.

Treatment	Time	ID	Ired	is.outlier	is.extreme
T	16	T17	658	TRUE	FALSE
T	45	T17	657	TRUE	FALSE

Table S15: Normal distribution of the dependent variable tested by Shapiro-Wilk normality test, related to STAR Methods.

Treatment	Time	variable	statistic	p
C	0	Ired	0.94	0.555
C	16	Ired	0.899	0.213
C	30	Ired	0.946	0.623
C	45	Ired	0.962	0.809
C	50	Ired	0.951	0.683
T	0	Ired	0.966	0.845
T	16	Ired	0.931	0.417
T	30	Ired	0.923	0.345
T	45	Ired	0.952	0.67
T	50	Ired	0.976	0.941

Table S16: Homogeneity of variance tested by Levene's test, related to STAR Methods.

Df	F	value	Pr(>F)
9	1.4646	0.1724	
95			

Table S17: Homogeneity of covariances tested by Box's M test, related to STAR Methods.

Statistic	p.value	parameter	Method
9.30	0.00230	1	Box's M-test for Homogeneity of Covariance Matrices

Table S18: ANOVA mixed model, effect of time and treatment on specimens' Ired values, related to Figure 4.

Effect	DFn	DFd	F	p	p<.05	ges
Treatment	1	19	2.79	0.111	0.095	
Time	4	76	1.694	0.16	0.025	
Treatment:Time	4	76	3.069	0.021	*	0.044

Post hoc test

Effect of the treatment factor -

Table S19: Effect of the treatment factor on every level of time, related to STAR Methods

Time	Effect	DFn	DFd	F	p	`p<.05`	ges	p.adj
0	treatment	1	19	0.006	0.939		0.00032	1
16	treatment	1	19	3.42	0.08		0.152	0.4
30	treatment	1	19	1.3	0.268		0.064	1
45	treatment	1	19	1.02	0.325		0.051	1
50	treatment	1	19	14.2	0.001	*	0.427	0.005

Table S20: Pairwise comparisons between treatment levels, related to Figure 4.

Time	group1	group2	n1	n2	p	p.signif	p.adj	p.adj.signif
0	C	T	10	11	0.939	ns	0.939	ns
16	C	T	10	11	0.0801	ns	0.0801	ns
30	C	T	10	11	0.268	ns	0.268	ns
45	C	T	10	11	0.325	ns	0.325	ns
50	C	T	10	11	0.00132	**	0.00132	**

Effect of the time factor –

Table S21: Effect of the time factor on every level of treatment, related to STAR Methods.

Treatment	Effect	DFn	DFd	F	p	`p<.05`	ges	p.adj
C	Time	4	36	2.03	0.11		0.074	0.22
T	Time	4	40	3.44	0.017	*	0.044	0.034

Table S22: Pairwise comparisons between time points at each treatment level, related to STAR Methods.

Treatment	group1	group2	n1	n2	p.adj	p.adj.signif
C	0	16	10	10	1	ns
C	0	30	10	10	1	ns
C	0	45	10	10	1	ns
C	0	50	10	10	0.441	ns
C	16	30	10	10	0.771	ns
C	16	45	10	10	1	ns
C	16	50	10	10	1	ns
C	30	45	10	10	1	ns
C	30	50	10	10	0.02	*
C	45	50	10	10	1	ns
T	0	16	11	11	0.092	ns
T	0	30	11	11	0.012	*
T	0	45	11	11	1	ns
T	0	50	11	11	0.123	ns
T	16	30	11	11	1	ns
T	16	45	11	11	1	ns
T	16	50	11	11	1	ns
T	30	45	11	11	1	ns
T	30	50	11	11	1	ns
T	45	50	11	11	1	ns
